# Biochemical response to substrate reduction therapy versus enzyme replacement therapy in Gaucher disease type 1 patients

**DOI:** 10.1186/s13023-016-0413-3

**Published:** 2016-03-24

**Authors:** Bouwien E. Smid, Maria J. Ferraz, Marri Verhoek, Mina Mirzaian, Patrick Wisse, Herman S. Overkleeft, Carla E. Hollak, Johannes M. Aerts

**Affiliations:** Department of Endocrinology and Metabolism, Academic Medical Centre, Amsterdam, The Netherlands; Department of Medical Biochemistry, Academic Medical Centre, Amsterdam, The Netherlands; Department of Biochemistry, Leiden Institute of Chemistry, Leiden University, Leiden, The Netherlands; Department of Bio-Organic Synthesis, Leiden Institute of Chemistry, Leiden University, Leiden, The Netherlands; Leiden Institute of Chemistry, Gorlaeus Laboratory, room number 0.3.15, Einsteinweg 55, 2300 RA Leiden, The Netherlands

**Keywords:** Gaucher disease, Eliglustat, Miglustat, Chitotriosidase, Glucosylsphingosine, Glucosylceramide, Enzyme replacement therapy

## Abstract

**Background:**

We retrospectively compared biochemical responses in type 1 Gaucher disease patients to treatment with glycosphingolipid synthesis inhibitors *miglustat* and *eliglustat* and ERT.

**Methods:**

Seventeen GD1 patients were included (*n* = 6 eliglustat, (two switched from ERT), *n* = 9 miglustat (seven switchers), *n* = 4 ERT (median dose 60U/kg/m). Plasma protein markers reflecting disease burden (chitotriosidase, CCL18) and lipids reflecting substrate accumulation (glucosylsphingosine, glucosylceramide) were determined. Also, liver and spleen volumes, hemoglobin, platelets, and fat fraction were measured.

**Results:**

In patients naïve to treatment, chitotriosidase, CCL18 and glucosylsphingosine decreased comparably upon eliglustat and ERT treatment, while the response to miglustat was less. After 2 years, median decrease of chitotriosidase was 89 % (range 77–98), 88 % (78–92) and 37 % (29–46) for eliglustat, ERT and miglustat naïve patients respectively; decrease of CCL18 was 73 % (63–78), 54 % (43–86), and 10 % (3–18); decrease of glucosylsphingosine was 86 % (78–93), 78 % (65–91), 48 % (46–50). Plasma glucosylceramide in eliglustat treated patients (*n* = 4) reached values below the normal range (*n* = 20 healthy controls). Biochemical markers decreased or stabilized in switchers from ERT to eliglustat (*n* = 2), but less in miglustat switchers (*n* = 7). Clinical parameters responded comparably upon eliglustat and ERT treatment.

**Conclusions:**

Our explorative study provides evidence that biochemical markers respond comparably in patients receiving eliglustat treatment and ERT, while the corresponding response to miglustat treatment is less.

## Background

Gaucher disease type I (GD1, OMIM230800) results from a deficiency of glucocerebrosidase (GBA1), a lysosomal enzyme responsible for the degradation of glucocerebroside (GlcCer) and glucosylsphingosine (GlcSph) [1, 2]. Subsequently, GlcCer and GlcSph accumulate, leading to the characteristic lipid laden macrophages, also known as Gaucher cells. These are thought to play a vital role in GD1’s pathophysiology, causing symptoms of hepatosplenomegaly, cytopenia and debilitating bone complications. Enzyme replacement therapy (ERT), currently the standard treatment of GD1, targets correction of these macrophages by intravenous administration of modified glucocerebrosidase. Three recombinant glucocerebrosidase products are approved for the treatment of GD1: imiglucerase (Genzyme a Sanofi Company), velaglucerase alpha (Shire Human Genetic Therapies) and taliglucerase alpha (Protalix Biotherapeutics). All demonstrate remarkable improvement of cytopenias and hepatosplenomegaly [3–8]. Nonetheless there are important limitations of ERT. Besides inconvenient infusions and exorbitant costs, there is increasing evidence that ERT cannot completely prevent bone complications [9]. In particular patients with bone disease at start of therapy can experience despite treatment additional bone complications, although the frequency of such events seems reduced [10]. It has been suggested that a systemic therapy targeting not only macrophages might be more beneficial, as aspects of GD’s symptomatology (malignancies, pulmonary hypertension, Parkinson’s disease and osteoporosis) are insufficiently explained by macrophage involvement solely [11].

Substrate reduction therapy (SRT), aiming to reduce accumulating glycosphingolipids by inhibiting their synthesis, might circumvent these disadvantages of ERT. Inhibitors of GlcCer synthesis to be used in SRT of GD1 are small compounds that can be taken orally and have the potential to rapidly diffuse into various tissues, including bones and the central nervous system. For GD1 presently inhibitors of the enzyme glucosylceramide synthase (GCS) have been developed. The first developed GCS inhibitor was miglustat (Actelion Pharmaceuticals Ltd.), which over a decade ago was approved for mild to moderately affected GD1 patients unsuitable to receive ERT. Significant improvements in hepatosplenomegaly and biochemical markers have been observed with miglustat treatment [12–14]. Although direct comparison with ERT has never been properly studied, the effects on key clinical parameters are less robust. Side effects such as gastrointestinal complaints (up to 80 %) and tremors in many cases have led to discontinuation of treatment. These side effects limit the use of miglustat for patients with GD [12, 13]. Although miglustat is able to cross the blood brain barrier in mouse models, effects on neurological outcomes in type III GD are controversial [15, 16]. Eliglustat tartrate (abbreviated to eliglustat, Genzyme a Sanofi Company) is a new GCS inhibitor with a stronger inhibitory potency than miglustat (IC50 value of 0.024 μM vs. 5–50 μM). Eliglustat does not cross the blood brain barrier. In contrast to miglustat, eliglustat does not potently inhibit intestinal glycosidases, thus largely preventing the gastro-intestinal symptoms observed with miglustat [17]. Eliglustat has shown high promise as an oral treatment for GD1 given the observed clinically relevant effects on hematological and visceral symptoms [18–22].

In GD1, well established plasma markers reflecting disease burden have been described. Chitotriosidase (*CHIT1* gene) and pulmonary and activation regulated chemokine (CCL18/PARC), both produced and secreted by macrophages, reflect to some extent the total burden of Gaucher cells [23, 24]. Chitotriosidase and CCL18 correlate with several clinical parameters [25, 26] and failures in correction of high levels of chitotriosidase are associated with the incidence of long term complications [26]. The use of plasma chitotriosidase in monitoring of GD1 patients has limitations, because chitotriosidase activity is subject to genetic heterogeneity. Roughly 6 % of the population has no chitotriosidase activity due to a 24-bp duplication in the CHIT1 gene [27, 28]. More importantly a common G102S CHIT1 polymorphism renders misleading data of chitotriosidase protein levels when using the commercial substrate 4-methylumbelliferyl-chitotrioside as a substrate [29]. This can be prevented using the novel 4-methylumbelliferyl-deoxychitobiosidase substrate [29, 30]. Even with the optimized 4-methylumbelliferyl-deoxychitobioside substrate the use of internal standards of recombinant chitotriosidase is warranted. Data produced by laboratories not using such internal controls should be interpreted with caution. In chitotriosidase-deficient GD patients CCL18 is frequently used to monitor GD1 disease. Little is known about intra-individual variations due to polymorphisms in the CCL18 gene. A more recent improvement is the use of plasma GlcSph as a marker of Gaucher cell burden. The sphingoid base is on average 200 fold elevated in GD1 patients [31]. The main source of the elevated GlcSph in GD1are lipid-laden macrophages, but all GBA deficient cells in GD patients may produce GlcSph locally [31]. In contrast to CCL18 and chitotriosidase, GlcSph is directly related to the primary molecular defect in GD1 patients. There is some evidence that GlcSph in GD1 is largely formed from intralysosomal GlcCer by deacylation [31–33]. Recent studies with conditional GD1 mouse models provide some evidence for the hypothesis that abnormalities in GlcSph contribute to GD1 symptomatology [11, 31, 32, 34, 35].

Until now a direct comparison of effects on biochemical markers reflecting disease burden between the aforementioned SRT and ERT treatment modalities has not been available. In this study, the effects on plasma markers of disease burden (chitotriosidase, CCL18, and GlcSph), plasma GlcCer associated to lipoproteins and clinical response (visceral, hematological and skeletal) are compared among eliglustat, miglustat and ERT treated patients.

## Methods

### Patients

All Dutch Gaucher patients treated with eliglustat and miglustat were eligible and included in this case series study. To compare clinical effects between treatment modalities, eliglustat treated patients (naïve to ERT therapy) were matched to ERT treated patients based on disease severity (absence of splenectomy and bone complications) and gender. Miglustat patients (naïve to ERT therapy) could not be matched due to small sample size. All ERT treated patients were receiving similar doses of ERT and had available fat fraction scores. Treatment failure was defined as published earlier [26]. A diagnosis of Gaucher disease was confirmed by GBA genotyping and demonstration of deficient glucocerebrosidase activity in leucocytes. Clinical assessments consisted of hemoglobin levels, platelets count and abdominal Magnetic Resonance Imaging (MRI) to assess liver and spleen volumes. Bone marrow infiltration was assessed using Dixon Quantitative Chemical Shifting Imaging (QCSI) of the lumbar spine [36].

In the Dutch cohort in total six patients received eliglustat as part of a trial program from Genzyme, a Sanofi company. All patients were treated with eliglustat, albeit in different dosing regimens and frequency (once or twice daily, see Table 1). Per protocol doses were adjusted based on plasma trough level of eliglustat. Five patients used 200 mg once or 100 mg twice a day, and one used 50 mg twice a day (patient 2).

**Table 1:** Baseline characteristics of included GD1 patients

Patient Gender Age start therapy [yr]			GBA	Mean dose ERT [U/kg/m] SRT [mg/day]	CYP2D6 Genotype	ERT before switch [yr]	Stop SRT/SRT duration [yr]	Anemia [g/dL] T_0_/T_Switch_	Platelets [^a^10^9^/L] T_0_/T_Switch_	Sx	Spleen [mL] T_0_/T_Switch_	Liver [mL] T_0_/T_Switch_	Bone complications	FF [%] T_0_/T_Switch_
Naive patients	Eliglustat	1 M 59	N370S/L444P	200	IM	na	-	12.6	76	-	888	2037	-	37
		2 M 42	N370S/L444P	100	IM	na	-	-	96	-	1700	2679	-	38
		3 M 43	N370S/L444P	200	EM	na	-	-	117	-	797	2728	-	29
		4 F 37	N370S/L444P	200	EM	na	-	-	154	-	642	2031	-	7
	ERT	5 M 47	N370S/L444P	45	-	na	na	-	118	-	855	2269	-	18
		6 M 45	N370S/P319L	60	-	na	na	-	69	-	2115	3347	-	33
		7 M 42	N370S/c.1265_1319del	60	-	na	na	13.5	78	-	508	1776	-	24
		8 F 46	RecNci/D140H	60	-	na	na	-	38	-	932	1733	-	44
	Miglu.	9 F 44	N370S/L444P	500	-	na	-	-	95	-	1412	2818	-	23
		10 F 43	N370S/R131C	300	-	na	-	11	268	+	na	2590	+	16
Switch patients	Eliglu.	11 M 37	N370S/L444P	15/200	EM	18	-	-/-	110/174	-	574/310	1830/1719	-	29/51
		12 M 36	N370S/G202R	34/200	EM	20	AE/0.33	12.6/12.7	281/281	+	na	2257/1327	+	na^a^
	Miglustat	13 M 43	N370S/L444P	15/300	-	12	-	-/-	45/162	-	885412	2014/1615	-	40/56
		14 M 47	N370S/L444P	34/300	-	13	-	11.3/-	53/100	-	3118/1855	3334/2193	-	24/52
		15 F 32	N370S/D409H	15/300	-	9	TF/0.73	11.1/-	41/155	-	766/232	2485/1832	-	7/45
		16 F 65	N370S/L444P	30/300	-	8	AE/0.23	11.9/-	76/192	-	1561/323	2730/1851	-	16/34
		17 F 12	N370S/L444P	30/300	-	11	AE/0.41	11.9/-	90/143	-	933/409	1682/1725	-	18/38
		18 F 30	N370S/EX9delG	30/300	-	7	TF/0.68	-/11.9	129/161	+	na	3293/1604	-	23/33
		19 F 23	N118S/N118S	30/200	-	2	TF/2.2	10.8/7.3	46/52	-	952/1557	2005/1878	-	4/4

Of the patients switching from ERT to SRT baseline data are given before start of ERT (T_0_) and before start of SRT (T_switch_). Dosing represents the mean dose of ERT/SRT during first two years of treatment. Anemia is defined as *M* < 13.7, *F* < 12.1 g/dL. ^a^ due to severe bone complications fat fraction cannot reliably be measured. Abbreviations: *M* males, *F* females, *IM* intermediate metabolizer, *EM* extensive metabolizer, *na* not applicable, +: present, -: absent, *ERT* enzyme replacement therapy, *SRT* substrate reduction therapy, *Sx* splenectomy, *AE* adverse events, *TF* treatment failure, *FF* fat fraction

A study protocol to obtain extra blood samples for patients treated with eliglustat was approved by the institutional review board of the Academic Medical Centre (AMC), Amsterdam, the Netherlands. Written informed consent was obtained from all participants. Data and samples of ERT and miglustat treated patients were gathered from the AMC biobank, for which all patients signed an informed consent. All investigations were conducted according to the declaration of Helsinki.

### Biochemical plasma markers

Biochemical markers were measured in non-fasted plasma samples, stored at -20 °C. Chitotriosidase activity was measured using the 4MU-deoxychitobiosidase substrate, as described [28, 30], using a recombinant chitotriosidase as internal standard. All chitotriosidase values of patients heterozygous for the 24-bp duplication in the CHIT1 gene were multiplied by 2 [27, 28]. Plasma CCL18 was determined by ELISA as described previously [23].

Glycosphingolipids were extracted as previously described using the Bligh and Dyer method. Briefly, 500 μL CHCl_3_/MeOH was added to 25 μL of plasma, in a 2:3 ratio (v:v), and samples were centrifuged for 3 min at 16 000 *g* to remove protein precipitate. Subsequently, 100 μL CHCl_3_ and 255 μL 100 mM formate buffer pH 3.15 was added for phase separation. After vortexing and centrifugation for 3 min at 16 000 *g*, the upper organic phase was collected, and lower phase re-extracted with 300 μL MeOH and 270 μL formate buffer. Pooled upper phases were desiccated in a heat block set at (37 °C) using mild N_2_ flow. Dried samples were redissolved in 100 μL MeOH and glucosylsphingosine was analysed by LC-ESI-MS/MS using ^13^C_5_-GlcSph as internal control [37] Ceramide and glucosylceramide were determined in the lower phase by HPLC as described in reference [38] using C17 sphinganine as an Internal Standard (Avanti Polar Lipids, Alabama, USA).

### Statistical analyses

For statistical calculations and data collection, SPSS version 20 was used (SPSS Inc. Chicago, IL). Graphs were made in GraphPad Prism version 6. For descriptive data, medians and ranges were used. Statistical tests to compare effects between groups were not performed due to small sample size.

## Results

### Patients

Of the included patients six were treated with eliglustat: four were naïve to treatment (‘naïve patients’) and two had been treated with ERT for 18 or more years (‘switch patients’). One switch patient (Table 1, no. 12), severely affected at initiation of ERT (multiple disabling bone complications, splenectomy), interrupted eliglustat treatment after 17 weeks because of an AE possibly related to eliglustat use (data reported to Genzyme a Sanofi Company). Nine miglustat treated patients were included of whom two were naïve to treatment. Seven patients switched from ERT to miglustat: three because of a worldwide imiglucerase shortage (no. 15, 16, 17), three because they preferred an oral treatment and were clinically stable on ERT (no. 13, 14, 18), and one because of treatment failure on imiglucerase due to development of neutralizing antibodies (no. 19, depicted in graphs with a blue star), earlier described in an abstract and case report [39, 40]. Two patients interrupted treatment because of tremors and three because of treatment failure (described more in detail below). The four patients treated with eliglustat and naïve to ERT were matched to four ERT treated patients (see method section and Table 1), receiving a median dose of 60 U/kg/month (range 45–60) albeit with different enzyme preparations (velaglucerase or imiglucerase).

### Chitotriosidase, CCL18 and GlcSph in patients naïve to treatment

**Fig. 1:**
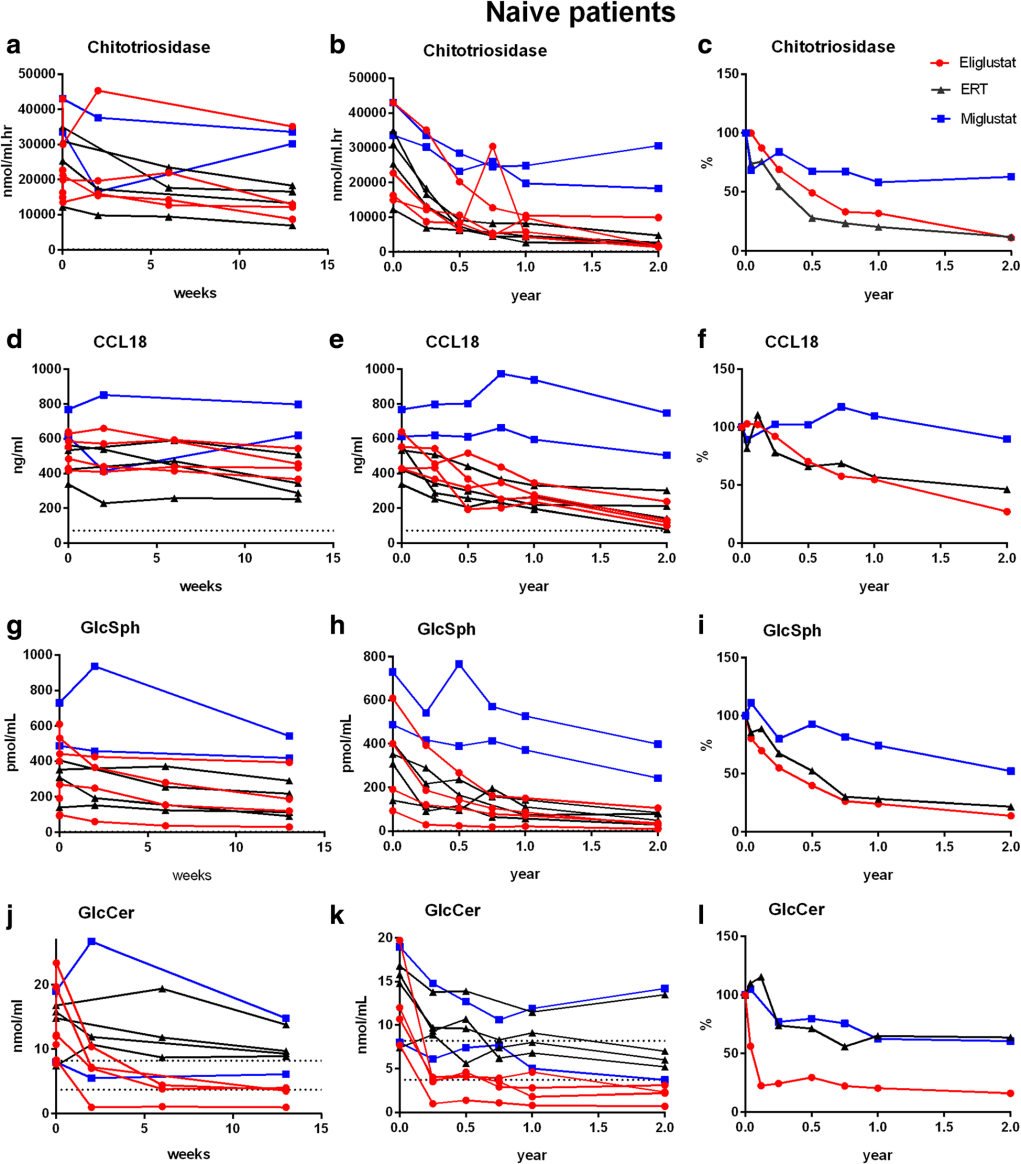
Plasma chitotriosidase, CCL18, glucosylsphingosine (GlcSph) and glucosylceramide (GlcCer) in patients naïve to treatment. Graphs **a**, **b**, **d**, **e**, **g**, **h**, **j**, **k**: represent absolute marker levels. Graphs **c**, **f**, **i**, **l**: represent median of individual relative levels compared to each individual T_0_ sample (100 %) per treatment modality. Dotted lines represent reference values (chitotriosidase: 7–187 nmol/mL.hr, CCL18: 10–72 ng/mL, GlcSph 0.8–2.7 pmol/mL, GlcCer 3.7–8.2 nmol/mL)

Figure 1 graphs A–F and Table 2 demonstrate that while plasma chitotriosidase, CCL18 and GlcSph levels decreased comparably in patients treated with eliglustat and ERT, in miglustat treated patients these decreases were less prominent (of note, one patient had bone complications and a splenectomy suggesting more severe disease). After two years of therapy none of the investigated markers normalized completely, irrespective of treatment modality. The reduction during the first three months of therapy of these markers was comparable between ERT and eliglustat treated patients.

**Table 2:** Median percent decrease (range) of biochemical markers in treatment naïve patients

		Eliglustat	ERT	Miglustat
		*n* = 4	*n* = 4	*n* = 2
	Year	Median (range)	Median (range)	Median (range)
Chitotriosidase	1	68.3 (40–80.9)	79.6 (61.9–91.1)	41.8 (41.4–42.2)
	2	88.8 (77–97.7)	88.1 (77.9–91.9)	37.3 (28.9–45.6)
CCL18	1	45.4 (35.3–52.9)	43.1 (21.5–65)	-9.6 (-22.1–2.9)
	2	72.9 (62.7–78.3)	53.7 (43.4–85.7)	10.3 (2.8–17.7)
GlcSph	1	75.9 (62.4–78.3)	71.9 (20.6–81.3)	25.8 (23.8–27.7)
	2	86.2 (78.4–92.9)	78.4 (64.7–90.6)	47.8 (45.4–50.2)
GlcCer	1	79.8 (76.4–89.4)	35.1 (-6.9–56.8)	37.6 (37.5–37.6)
	2	84.1 (74.3–90.6)	36.4 (19–67.1)	39.5 (25.3–53.6)

### Chitotriosidase, CCL18 and GlcSph in patients switching from ERT to SRT

**Fig. 2:**
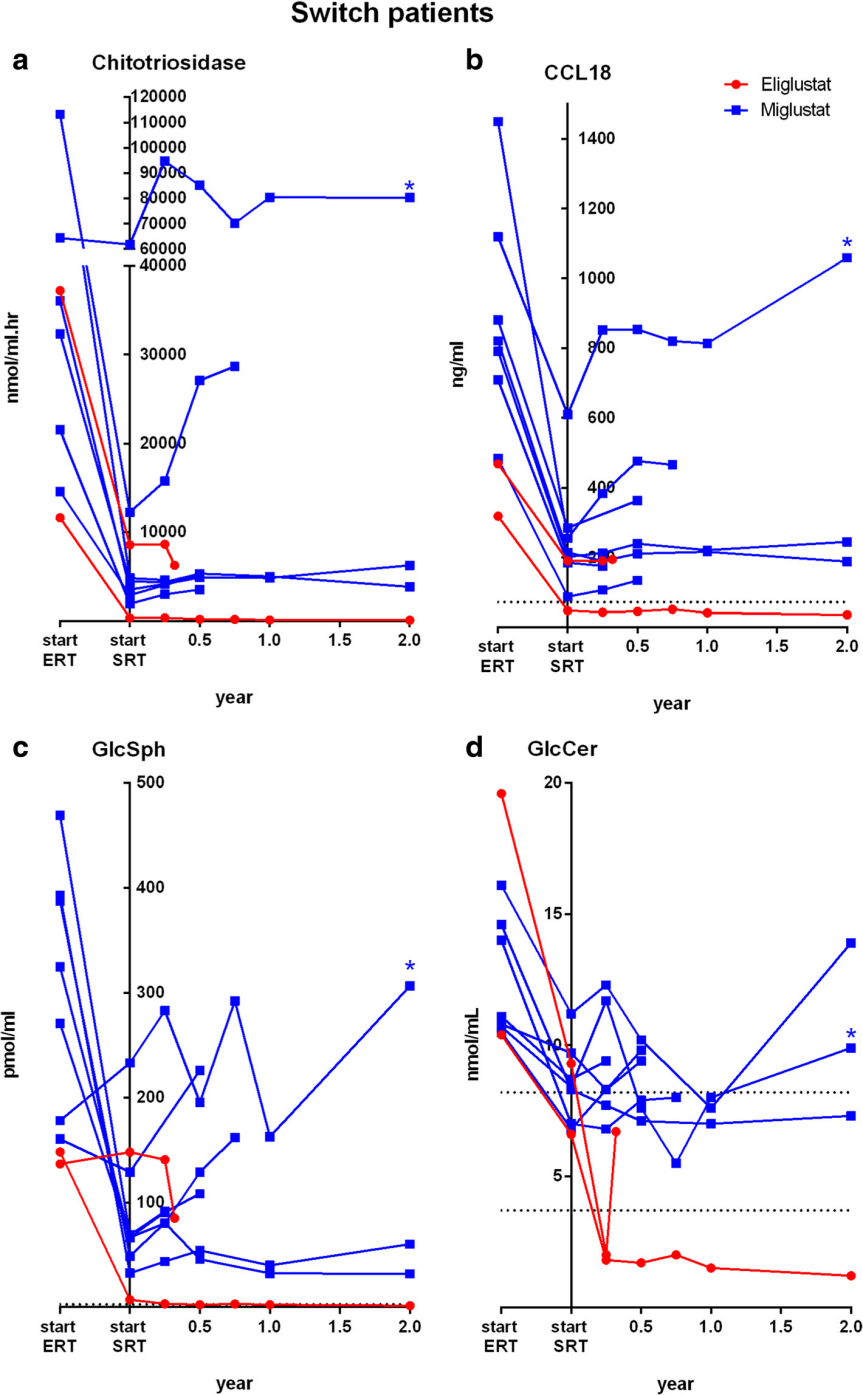
Plasma chitotriosidase, CCL18, glucosylsphingosine (GlcSph) and glucosylceramide (GlcCer) in patients previously treated with ERT. **a**–**d**: individual levels. Dotted lines represent reference values (see legend Fig 1). Time between start ERT and SRT is variable between patients (range 7–13 year). * *blue star* indicates patient no 19

Only two patients switching from ERT to eliglustat were available. Patient 11, a mildly affected patient switching from ERT to eliglustat, demonstrated a further decrease of chitotriosidase and GlcSph while CCL18, already normalized on ERT, remained constant. Despite the fact that patient 12 was only treated with eliglustat for a short period of time (17 weeks), he demonstrated a decline in chitotriosidase, which was still very high after 20 years of ERT (8572 nmol/mL.hr). Of note, this patient also showed a transient reduction in plasma GlcSph. In patients switching from ERT to miglustat, plasma chitotriosidase (5/7), CCL18 (5/7) and GlcSph (6/7) increased in most patients in comparison to their levels before switch (Fig 2a–d). At the last available sample before discontinuation (combined with the sample after two years of treatment in those not interrupting treatment, sample range 0.23 to 2 years), chitotriosidase increased with a median of 78 % (range -14 to +134), CCL18 28 % (range -13 to +83 %) and GlcSph 63 % (range -34.7 to +143 %).

### Plasma GlcCer and Ceramide in patients naïve and patients switching from ERT to SRT

To compare the inhibitory response of eliglustat and miglustat on GlcCer synthesis at cellular level, tissue biopsies would have been essential. Because these were not available we investigated plasma GlcCer levels, mostly reflecting hepatic GlcCer synthesis bound to lipoproteins, and spill-over of accumulated GlcCer. Plasma GlcCer levels decreased rapidly below lower limit of normal (of *n* = 20 healthy control subjects), both in naïve and switchers treated with eliglustat (Figs. 1j and 2d). Decreases were observed already after two weeks of treatment (data not shown). Such rapid decline of plasma GlcCer and GlcSph was not observed in any of the miglustat treated patients. Of note, we observed an unexpected transient increase of GlcCer and GlcSph in 5/6 patients in samples taken 4 h after their first dose of eliglustat (data not shown). Whether this unexplained effect is specific to eliglustat could not be investigated as short term samples were not available from ERT and miglustat treated patients. In contrast, in most miglustat treated patients, especially those who switched from ERT (5/7), plasma GlcCer increased to supra-normal levels. Upon ERT treatment plasma GlcCer levels decreased in all, but normalized in 3/4 patients treated with ERT only, and normalized in 2/7 switch patients just before they switched to miglustat.

Plasma ceramide levels did not increase upon different treatment modalities in naïve or switch patients: T_0_ eliglustat 6.6–12.8 nmol/mL, ERT 8.0–15.0 nmol/mL, miglustat 6.6–10.6 nmol/mL (normal reference range: 5.1–18.8 nmol/mL) versus after two years of treatment T_2yr_ eliglustat 7.6–12.8 nmol/mL, ERT 8.0–10.0 nmol/mL, miglustat 4.8–10.1 nmol/mL.

## Clinical response

### Eliglustat

**Fig. 3:**
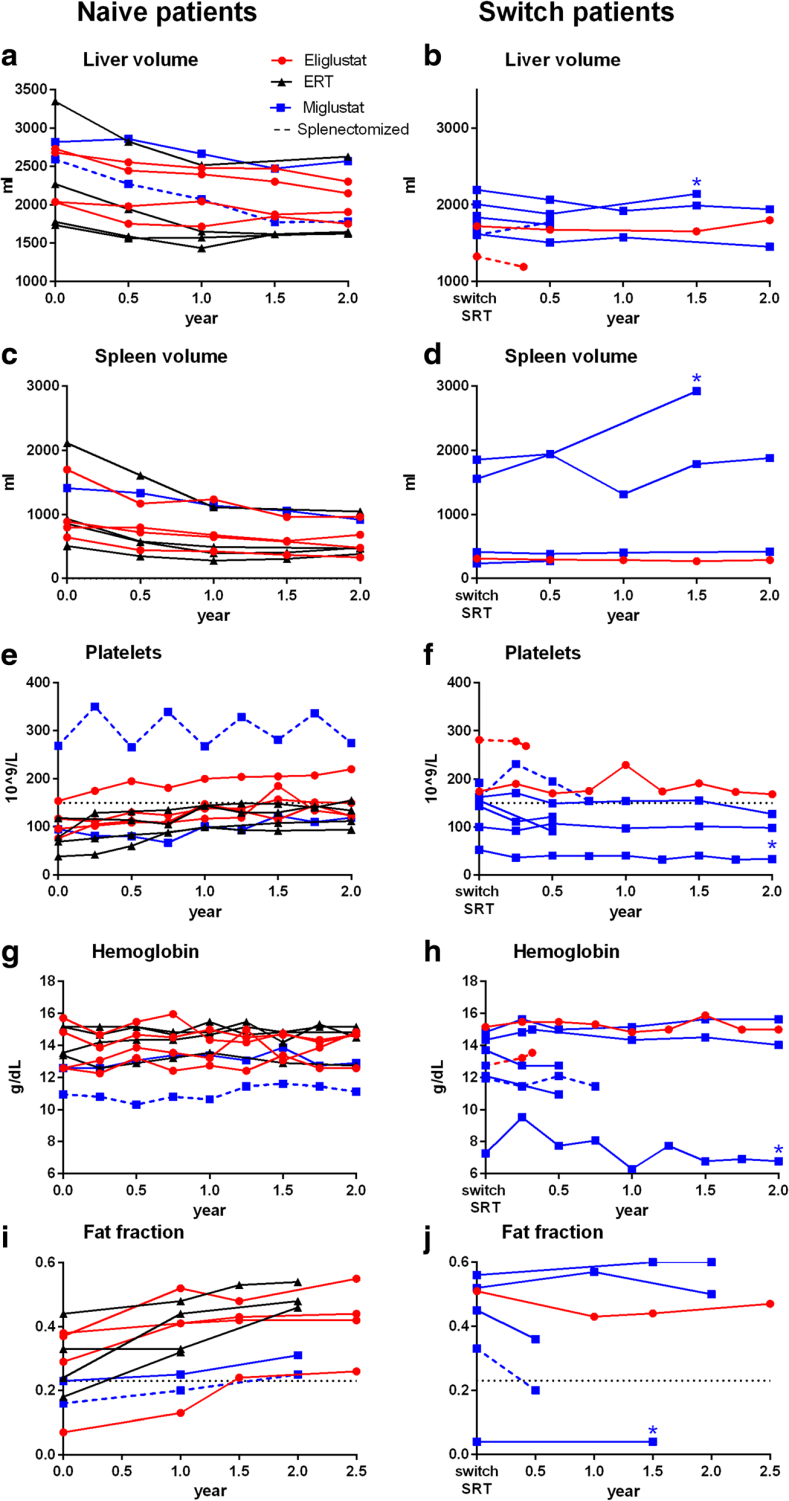
Clinical response of different treatment modalities in naïve patients (graphs ACEGI) and switching from ERT to SRT (graphs BDFHJ). Follow up of patients 15 and 16 is incomplete due to early treatment discontinuation. **a**, **b** Liver volume. **c**, **d** Spleen volume. **e**, **f** Platelet count, splenectomized patients are depicted in dotted lines. **g**, **h** Hemoglobin levels, splenectomized patients are depicted in dotted lines. **i**, **j** Average bone marrow fat fraction of corpora L3, L4, L5 (data are missing of patient 12. Due to multiple bone complications fat fraction assessments are not reliable, patient 16 and 17 because of early treatment discontinuation). Below the indicated threshold value of 0.23 (*black dotted-line*), the risk for bone complications is increased. * *blue star* indicates patient 19

**Fig. 4:**
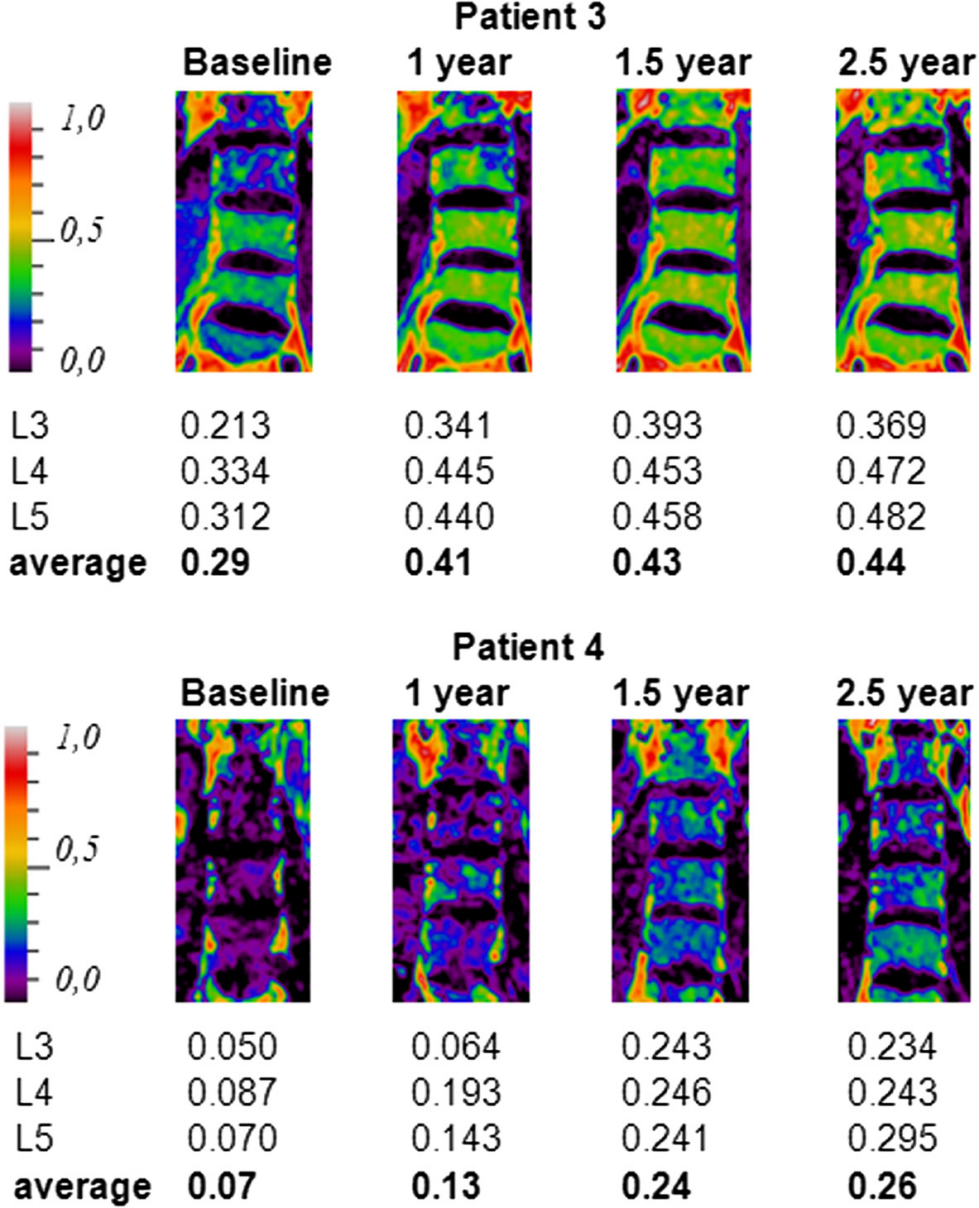
Changes in bone marrow fat fraction during 2.5 years of eliglustat treatment. Depicted are patient no. 3 and 4 who had the most severe decrease of fat fraction before initiation of therapy and showed most prominent increases of fat fraction upon eliglustat treatment. Fat fraction values of patient 4 before initiation of therapy ranged between 7–20 %, probably related to hormonal variation [53]. Fat fraction scores of L3, L4, and L5 are averaged. Patients with a fat fraction <0.23 have an increased risk of developing bone complications [36]

Eliglustat treated patients naïve to treatment were mildly affected by GD1 at initiation of therapy. Figure 3 demonstrates the good clinical response to the drug treatment: liver and spleen volumes decreased, platelet counts increased and no bone complications occurred. Hemoglobin levels improved in those with anemia at baseline. Bone marrow fat fraction levels increased in all patients upon eliglustat treatment: three normalized their fat fractions by one year and the fourth increased above a threshold (0.23) associated with an increased risk of fractures [36]. These improvements are indicative of clearance of Gaucher cells from the bone marrow compartment (Figs. 3i and 4).

Of the two switch patients, one (no. 12) was a severely affected patient with splenectomy and multiple bone complications prior to initiation of ERT. Twenty-two years of ERT had resulted in a normal liver volume, but residual bone disease with high plasma chitotriosidase levels. He interrupted treatment with eliglustat after 17 weeks due to an AE. All clinical GD parameters remained stable, but the period of observation was too short to draw any conclusions. Patient 11, mildly affected at start of ERT, had stable spleen and liver volumes, platelets counts and hemoglobin levels, which were all normal when eliglustat was initiated and remained stable.

### Miglustat

Miglustat patients had variable disease severity at initiation of ERT. Of the two patients treated with miglustat and naïve to ERT, one splenectomized patient with bone complications (no. 10), showed a gradual decline of liver volume, comparable to ERT and eliglustat patients (Fig. 3a). After 15 years of treatment this patient kept borderline anemia (Hb levels T_0_ 10.9 to T_15_ 10.9 g/dL) without other causes for her anemia than GD1. The non-splenectomized patient had a decline in organomegaly, which was similar to effects seen with ERT and eliglustat during the follow-up of two years. Evaluation after 15 years of treatment showed stabilization with moderate thrombocytopenia (100 × 10^9^/L) and splenomegaly (406 mL, 3 times multiple of normal). Fat fractions increased in both patients (Fig. 3i).

Of the seven patients switching to miglustat two remained clinically stable (no. 13 and 14). Two patients discontinued treatment shortly after initiation of miglustat due to AEs and three patients discontinued due to treatment failure (see Table 1). The first had an increase of spleen volume and a decrease of fat fraction, while the second had an increase of liver volume and decrease of fat fraction The third case (patient 19) was switched to miglustat due to disease progression as the consequence of neutralizing antibodies to imiglucerase. She further deteriorated on miglustat treatment with an increase of liver volume, development of severe thrombocytopenia and a bone complication with very low fat fraction (see blue star * in Figs. 2, 3). In all patients who had a treatment failure, the biochemical markers chitotriosidase and CCL18 worsened upon start of miglustat treatment.

## Discussion

Our investigation with a limited number of naïve GD1 patients suggests that the response to eliglustat treatment with respect to established biomarkers of disease burden (chitotriosidase and CCL18) is on a par with that of moderate doses of ERT. In contrast, the same biomarkers respond less favorably to miglustat treatment. These findings are in agreement with literature reports. Mistry et al demonstrated that after 9 months of eliglustat treatment in naïve GD1 patients chitotriosidase decreased with a mean of 44 % [21]. Lukina demonstrated a median decrease of CCL18 of 50 % [19], while in naïve GD1 patients treated with miglustat mean decreases of chitotriosidase after one year were less pronounced, ranging from 6 to 17 % [12, 41–44], median 5–13 % (median values were not given, but could be calculated from references [12, 42]).

Next, our investigation showed that seventy percent of patients switching from ERT to miglustat demonstrated an increase of plasma chitotriosidase levels. The prevalence of such deterioration is higher than reported in literature, where increases are described in 4–43 % of cases [13, 44–46]. The small number of patients investigated might explain this disparity. There may also be bias in studies due to high drop-out rates by AE’s and treatment failure. In addition, the outcome of the switch from ERT to miglustat may be influenced by specific reasons, for instance the severely affected patient no 19 in our investigation had to switch due to antibody development towards imiglucerase. Since increases of plasma chitotriosidase are not always directly accompanied by prominent clinical deterioration [45], the value of chitotriosidase measurements to monitor GD1 patients deserves discussion. Recently van Dussen et al demonstrated that patients with sustained chitotriosidase increases ≥ 30 % are at risk of having clinical deterioration with a relative risk of 6.3 (CI 95 % 2.2–17.8): chitotriosidase increases are accompanied by clinical deterioration in 50 % of patients, whereas when chitotriosidase is stable, 8 % develop clinical deterioration [26]. Sustained increases of chitotriosidase after switch from ERT to miglustat reflect an increase of Gaucher cell burden and risk for clinical deterioration or new complications, which should therefore be avoided. In addition, van Dussen showed that high residual chitotriosidase activity after two years of treatment correlates to long term complications. Although limited patients switching from ERT to eliglustat were available in this study, it is reassuring that even after ±3 months of therapy in a severely affected GD1 patient with high residual chitotriosidase activity after 20 years of ERT, eliglustat was able to decrease chitotriosidase and CCL18 levels. Even more promising is the noted transient reduction in plasma GlcSph in this patient. This anecdotal finding suggests that eliglustat might be capable in some GD1 patients to reach Gaucher cells that are not responsive to ERT. Our investigation documents for the first time that elevated plasma GlcSph is comparably decreased by eliglustat and ERT in naïve GD1 patients. The two investigated patients who switch from ERT to eliglustat treatment showed a further reduction of plasma GlcSph levels. On the other hand, miglustat treatment of naïve GD1 patients led to only minor reductions and switching patients from ERT to miglustat tended to be associated with stable or increasing plasma GlcSph. The clinical implications of the elevated plasma GlcSph are yet unclear, like those of elevated plasma GlcCer. From the single study that addressed associations with clinical symptoms, it can be concluded that it could be seen as a general marker for disease burden in GD1 [31], but does not correlate to specific symptoms. In contrast to chitotriosidase, which is not pathogenic but a mere reflection of alternatively activated macrophages, it has been hypothesized that GlcSph as a toxic compound is directly implicated in GD1 pathology. Studies of Orvinsky and Nilson revealed that GlcSph levels were normal in a brain of a GD I patient, but elevated in GD III and highest in brains of GD II patients with the most severe cerebral involvement [47, 48]. In vitro studies suggest neurotoxicity of GlcSph [49]. In addition, GlcSph has been shown to cause hemolysis and to inhibit protein kinase C, a pivotal kinase in signal transduction and cell behavior [31]. Extrapolation of such in vitro findings with high concentrations of GlcSph to pathophysiological action in GD patients warrants care. Further circumstantial evidence of a toxic effect of GlcSph in GD1 has been offered by Mistry et al demonstrating that high levels of GlcSph impair osteoblastogenesis in cultured osteoblasts of a conditional mouse GD1 model demonstrating very low bone mineral density [11]. More recently, Pavlova et al suggested that GlcSph is oncogenic, based on the association of high GlcSph levels in a conditional mouse model with a high incidence of B-cell lymphoma [34, 35]. If further research substantiates that GlcSph contributes to the pathogenesis of GD1, ERT treated patients with remaining high levels of GlcSph might potentially benefit from eliglustat treatment.

Of note, our study confirms that eliglustat is a very potent inhibitor of GCS in humans. Whereas with miglustat plasma GlcCer normalized or increased, eliglustat dramatically decreased circulating GlcCer bound to lipoprotein in all eliglustat patients, even to levels 50 % below normal as determined for 20 healthy subjects. However, it is essential to analyze a much larger number of presumed normal subjects to establish the lowest limit of the normal population range.

Due to limited sample size our study is insufficient to draw definite conclusions whether ERT and eliglustat are on a par in clinical efficacy. Nonetheless, our data for the matched patients indicate that eliglustat is not inferior to moderate dose of ERT: an equal decline of liver and spleen volumes, and equal rise of platelets and fat fraction was observed. Extended comparative data on both treatment modalities are now essential to determine the optimal clinical strategy for naïve GD1 patients. Such data are not actively pursued. The presently conducted RCTs by Genzyme do not directly compare ERT to eliglustat treatment of naïve GD1 patients. Instead, eliglustat treatment is compared to placebo [21]. Safety of switching from ERT to eliglustat treatment is separately investigated [22]. The RCT comparing placebo to eliglustat, including mild to moderately affected patients (severe patients with bone disease and splenectomies were excluded), demonstrates clinically relevant responses on cytopenia and hepatosplenomegaly [21]. Results of the ENCORE trial [22] demonstrate that 85 % (84/99) of the eliglustat treated GD1 patients (pretreated with ERT) and 94 % (44/47) of imiglucerase treated patients maintained therapeutic goals. Eliglustat treatment was statistically non-inferior to imiglucerase treatment as the lower bound of the 95 % confidence interval difference was within pre-specified non-inferiority thresholds (-17.6 %).

Potential advantages of present and future SRT modalities are thought to be better prevention of bone complications due to drug delivery in the bone compartments. In our naïve GD patients, bone marrow infiltration (fat fraction) improved upon eliglustat therapy, and none developed bone complications during the study period. With the available limited clinical data is not possible to draw conclusions on superiority of eliglustat treatment over ERT regarding bone complications. It will be also of interest to learn how eliglustat treatment impacts the occurrence of long-term complications and associated conditions in GD1 such as cancer, pulmonary hypertension, metabolic alterations including insulin resistance as well as Parkinson’s disease [50–52].

## Conclusions

Our exploratory investigation with a small number of GD1 patients has indicated that response in biomarkers of disease burden is on a par in naïve patients receiving eliglustat treatment and those receiving moderate doses of ERT, the same biomarkers responded less to miglustat treatment in (very limited sample size). Clinical response in the compared ERT and eliglustat treated naive GD patients seemed also comparable. Our present biochemistry and clinical findings should be extended by studies with larger cohorts.
